# Platypnea-Orthodeoxia Syndrome Secondary to Patent Foramen Ovale and Pulmonary Thromboembolism

**DOI:** 10.7759/cureus.79082

**Published:** 2025-02-16

**Authors:** Ana Sara Monteiro, Rita Rosa Domingos, Sofia R Amalio

**Affiliations:** 1 Internal Medicine, Unidade Local de Saúde do Algarve - Hospital de Faro, Faro, PRT

**Keywords:** aortic diseases, paradoxical embolism, patent foramen oval, platypnea-orthodeoxia, pulmonary embolism (pe)

## Abstract

Platypnea-orthodeoxia syndrome (POS) is a rare condition characterized by dyspnea (platypnea) and hypoxemia (orthodeoxia) in the upright position, with symptom relief in the supine position. It is often linked to right-to-left shunts, such as a patent foramen ovale (PFO), and other factors like pulmonary embolism (PE) and aortic dilation. We present an 86-year-old woman with hypertension and dementia who developed POS following multiple events. She previously experienced platypnea after a vaginal hysterectomy 4 years before the present event, which resolved with hemoglobin normalization. On admission for ischemic stroke, she developed hypoxemia upon standing, which improved in the left lateral decubitus position. Pulmonary CT angiography revealed PE, and transthoracic echocardiography identified a left-to-right intracardiac shunt. Transesophageal echocardiography confirmed a PFO with bidirectional flow. Despite the diagnosis, surgical closure was not pursued due to her comorbidities, and she was treated with anticoagulation and oxygen therapy. This case highlights the diagnostic challenges of POS, which can result from a combination of intracardiac (PFO), pulmonary (PE), and aortic causes. The patient’s symptoms were initially misattributed, in the first event of platynea that occurred 4 years before, delaying diagnosis. Paradoxical embolism from PFO may have contributed to her ischemic stroke. Early recognition of POS could improve outcomes, preventing complications like stroke and respiratory failure. POS is often underrecognized, especially in patients with unexplained positional dyspnea and stroke. Timely diagnosis and management can reduce the risk of severe complications, including paradoxical embolism, but a high degree of suspicion is necessary.

## Introduction

Platypnea-orthodeoxia syndrome (POS) is a rare condition first described in 1949. It is characterized by the presence of dyspnea in orthostasis (platypnea) and hypoxemia (orthodeoxia) with symptomatic relief and improvement in partial pressure of oxygen (PO2) in the supine position. This desaturation is defined by a drop in PO2 >4 mmHg or peripheral O2 saturation >5% when changing from the supine to the standing position [1].

The exact prevalence is unknown, but since its discovery, POS has been described in association with several diseases in more than 200 patients [1,2].

The pathophysiology responsible for this syndrome, although not fully understood, is associated with the mixing of deoxygenated venous blood with arterial blood through intracardiac and/or pulmonary shunts. Intracardiac shunts may be due to atrial septal defects, fenestrated atrial septal aneurysms, and patent foramen ovale (PFO). Age-related degenerative changes - such as aortic root dilation, elongation of the ascending aorta, and kyphoscoliosis - can alter the anatomical relationship between the atrial septum and venous blood flow, triggering POS later in life. These changes promote a preferential right-to-left blood flow through a PFO, particularly in the upright position, leading to hypoxemia and dyspnea. In turn, intrapulmonary shunts may be due to purely vascular alterations (arteriovenous malformations, pulmonary thromboembolism, hepatopulmonary syndrome), parenchymal alterations (interstitial diseases), and a mixture of both, conditioned by a ventilation-perfusion mismatch [2].

PFO is present asymptomatically in 30% of the general population. Right-to-left intracardiac shunt due to the presence of a PFO was the most frequently described structural anomaly associated with POS. The positional change to an orthostatic position leads to stretching of the interatrial septum, favouring the flow of venous blood directly from the inferior vena cava to the left atrium, with consequent dyspnea and hypoxemia in the sitting or orthostatic position [3].

POS remains an underdiagnosed yet potentially reversible cause of dyspnea, particularly in elderly patients with structural heart changes. This case highlights how an initially asymptomatic anomaly, such as a PFO, can become clinically relevant in later life due to degenerative conditions, like aortic dilation, and pulmonary embolism, leading to intracardiac pressure imbalances and the onset of platypnea and orthodeoxia. Recognizing POS is critical, as timely intervention - such as percutaneous PFO closure - can significantly improve a patient’s quality of life and prevent further complications.

Given its rarity and atypical presentation, POS should be considered in patients with unexplained positional dyspnea, particularly when hypoxemia worsens in an upright position and improves when supine. A high index of suspicion, coupled with appropriate diagnostic imaging, is essential for accurate diagnosis and effective treatment.

## Case presentation

We present the case of an 86-year-old woman with a history of hypertension and dementia syndrome. Her medical records indicate a hospitalization four years earlier due to severe bleeding following a vaginal hysterectomy. During that episode, she developed anemia from significant blood loss (hemoglobin 7 mg/dL) and experienced positional dyspnea, with worsening in the sitting position, associated with hypoxemia (oxygen saturation (SO2) 82% on fraction of inspired oxygen (FiO2) 80%). Her oxygen saturation improved when supine (SO2 96%). At the time, POS was suspected, prompting a transthoracic echocardiogram and a pulmonary CT angiography. The echocardiogram revealed dilation of the ascending aorta (46 mm) and an interatrial septum that appeared continuous (shunt evaluation was not performed at that time), while the CT angiography showed no significant findings, including no evidence of pulmonary embolism. The patient’s symptoms resolved completely after normalization of hemoglobin levels, during hospitalization. No further investigations were pursued at that time

**Figure 1:**
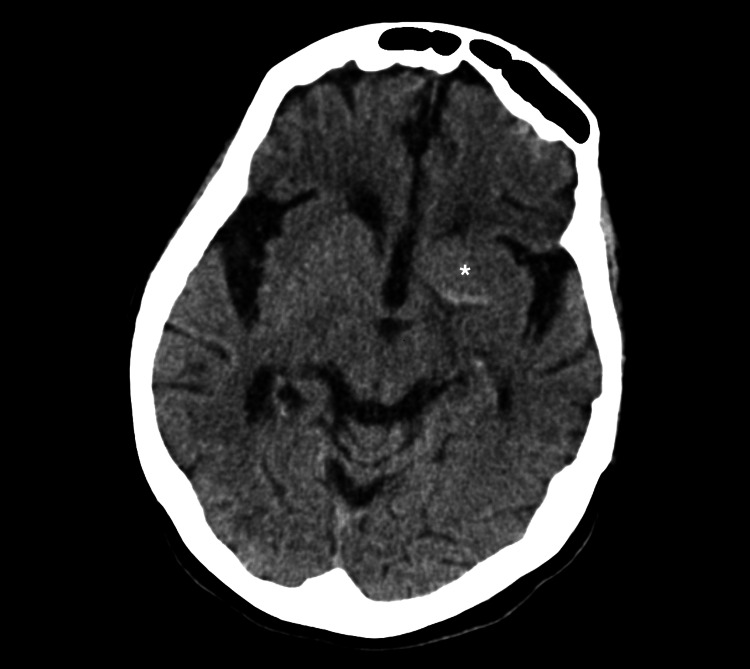
Cranial CT angiography. Absence of contrast column filling with hyperdensity of the left proximal middle cerebral artery suggesting endoluminal thrombus in the M1 segment (*).

Four years later, the patient was admitted to the hospital emergency department due to the sudden onset of global aphasia, right hemiplegia, left oculocephalic deviation, and right homonymous hemianopsia with altered facial expressions, scoring 24 on the National Institutes of Health Stroke Scale (NIHSS). She was hypertensive (160/80 mmHg), with a normal heart rate, euglycemic, without the need for oxygen therapy, and the electrocardiogram was in sinus rhythm. Computed tomography angiography (CT angiography) showed hyperdensity of the supraclinoid internal carotid artery and the left proximal middle cerebral artery, suggesting endoluminal thrombus in the M1 segment (Figure 1).

The diagnosis of cortico-subcortical ischemic stroke in the left ascending parietal circumvallation with thrombus in M1 was admitted, and the patient underwent fibrinolysis with partial resolution of the deficits (NIHSS 2). One day after the ictus the patient was started on prophylactic anticoagulation with low molecular weight heparin (40 mg daily). On the third day of hospitalization in the stroke unit, the patient presented hypoxemic respiratory failure after standing up, with improvement in the left lateral decubitus position (Table 1). Blood tests showed N-terminal type B natriuretic peptide and high sensitivity troponin within the reference ranges.

**Table 1:** Arterial blood gas analysis in different positions. FiO_2 _- fraction of inspired oxygen; pO_2_ - partial pressure of oxygen; pCO_2_ - partial pressure of carbon dioxide; SO2 - oxygen saturation; HCO3 - bicarbonate

	FiO_2 (_%)	pH	pO_2_ (mmHg)	pCO_2_ (mmHg)	SO_2_(%)	HCO_3 _mmol/L
Sitting at 45º	90	7.47	41.1	22.7	51	20.1
Supine	40	7.43	78.7	36.6	95	23.7

**Figure 2:**
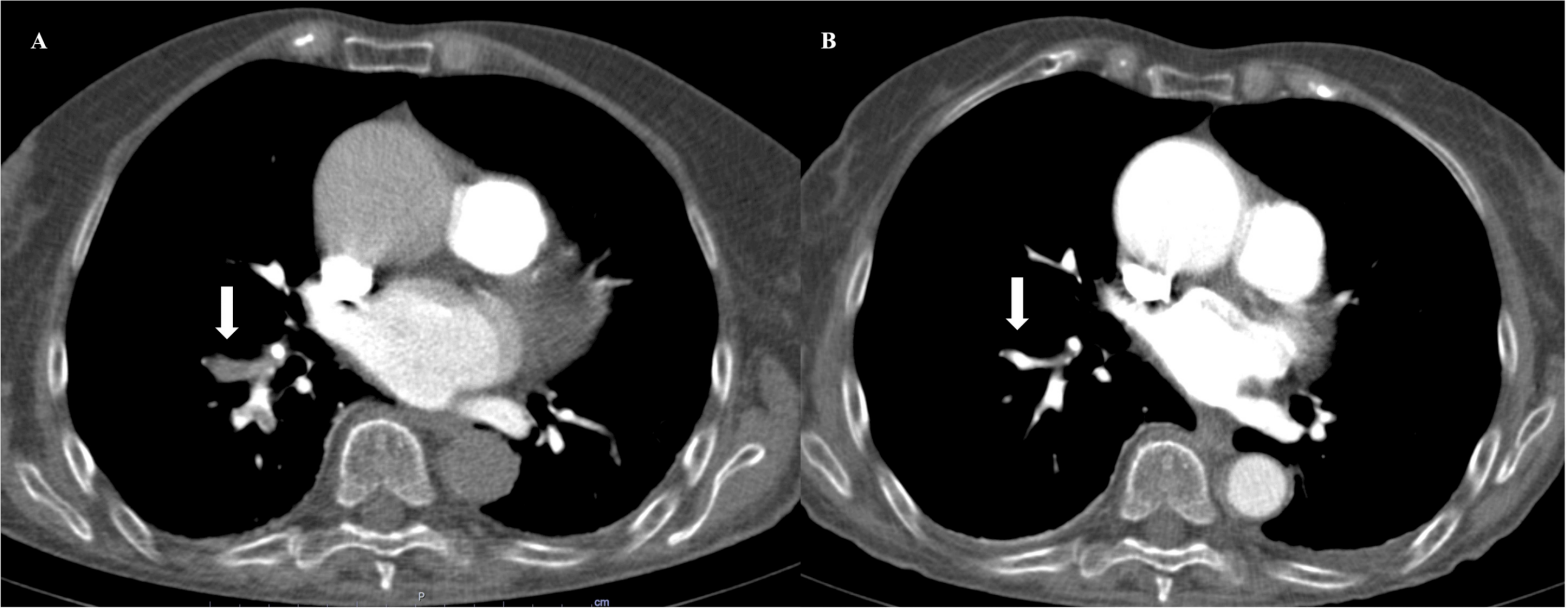
CT angiography of the chest on the third day of hospitalization. CT angiography of the chest on the third day of hospitalization (A) showing PE in the anterior, lateral, and posterior segmental branches of the right lower lobe (arrow). (B) Seven days after the start of hypocoagulation, permeabilization was observed in the segments where the absence of opacification had previously been identified (arrow).

Pulmonary CT angiography was performed, which showed filling defects in the segmental and subsegmental branches of the right upper and lower lobe, related to pulmonary embolism (PE) (Figure 2A).

**Figure 3:**
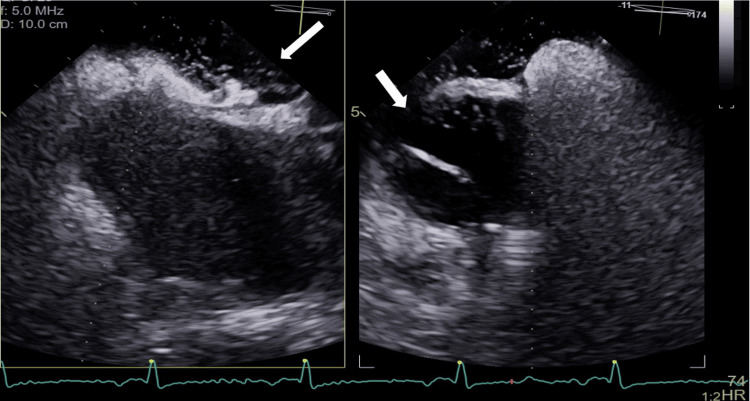
Transesophageal echocardiogram. Interatrial septum with patent foramen ovale (arrow) (A). Right-to-left shunt after administration of agitated saline solution, with passage of more than 30 bubbles in the first 3 cycles (B).

Ectasia of the ascending aorta (42 mm) was also observed. Doppler echocardiography of the lower limbs identified the presence of a partial thrombus in the right popliteal vein, causing reduced vascular filling. The patient was started on anticoagulation with low molecular weight heparin (1 mg/kg 12/12h). Two weeks after the PE, she continued to complain of positional dyspnea, which worsened in the sitting position, and was relieved in the left lateral decubitus position. Repeated CT angiography demonstrated permeabilization of the previously affected segments (Figure 2B). Transthoracic echocardiography showed a left-to-right shunt proximal to the mitral-tricuspid valve apparatus. Given the previous findings and the suspicion of a PFO, transesophageal echocardiography was performed, which confirmed the presence of an interatrial septum with PFO with bidirectional flow and dilation of the ascending aorta of 48-49 mm (Figure 3).

Given the patient's comorbidities and previous degree of dependence, surgical correction of the PFO was not performed. After the rehabilitation program, the patient was discharged home with therapeutic oral anticoagulation and long-term oxygen therapy (FiO2 36%) with a progressive reduction to FiO2 28%.

## Discussion

As a rare condition, POS is a diagnostic challenge. The presence of platypnea and hypoxemia in the orthostatic position should raise this suspicion.

The present case combines several factors that favor the development of POS: the existence of intracardiac causes (absence of the foramen ovale closure) and extrinsic causes such as dilatation of the ascending aorta. The presence of aortic abnormalities as root dilatation and elongation has been proposed to contribute to a right to left shunt in the presence of structural defects such as a PFO. The latter allows abnormal communication of blood flow, especially in the upright position. This is due to anterior and inferior displacement of the aortic root, due to gravity, increasing its size, decreasing the atrial septum size, and increasing its mobility. As a result, the loose atrial septum acts as a “spinnaker effect” in venous blood flow, keeping the PFO open. Additionally, a rightward shift of the fossa ovalis and narrowing of the angle between the atrial septum and inferior vena cava brings venous blood flow closer to the septal defect and compression of the right atrium by the aortic root may decrease its compliance, further facilitating shunting [1, 4]. These degenerative structural changes contribute to right-to-left shunting and the development of POS in elderly patients. We believe that the presence of pulmonary vascular causes (pulmonary thromboembolism) contributed in a subtle manner. This is because, despite the absence of increased right atrial pressure observed in the echocardiogram, the symptoms related to POS started at the same time as PE. 

The presence of PFO was without clinical relevance until the age of 82 when the patient presented hemorrhage after surgery. At that time, anemia unmasked a pre-existing right-to-left shunt by lowering oxygen and for the first time, the patient manifested symptoms of orthopnea and platypnea. Since the patient presented symptomatic resolution after stabilization of the hemoglobin values and no septal defect was observed in transthoracic echocardiography, the symptoms were no longer interpreted as such. At the age of 86, the presence of an ischemic stroke due to paradoxical embolization, therefore cardioembolic, appeared as the first manifestation of this anomaly. The prevalence of PFO in patients with cryptogenic stroke, as in the present case, is higher when compared to the general population [5]. According to the Risk of Paradoxical Embolism score (RoPE) [6], the patient presented a 38% chance that the stroke was due to PFO. The occurrence of embolization of a thrombus from the lower limb to the pulmonary tree, giving rise to a PE, may have led to an increase in pulmonary vascular resistance, albeit in a subtle manner, considering that the probability of pulmonary hypertension was low in the echocardiogram. This, in turn, conditioned the change in the intracardiac pressure gradient, leading to an increase in the intracardiac right-to-left shunt. Finally, the presence of aortic dilation, a degenerative change associated with age, interfered with the positioning of the atrial septum, bringing the vena cava blood flow closer to the PFO facilitating the shunting [7].

## Conclusions

Platypnea-orthodeoxia syndrome is a rare condition requiring high clinical suspicion, often linked to various pathologies. Symptoms of PFO may remain silent from birth and manifest later in life due to acute illness, hemodynamic changes and degenerative disease as aortic dilation. A right-to-left shunt, exacerbated by postural changes, leads to hypoxemia.

Early diagnosis of this condition, at the time of the first clinical presentation, would have allowed closure of the PFO preventing complications and improving outcomes.
